# Peptide YY Causes Apathy-Like Behavior via the Dopamine D2 Receptor in Repeated Water-Immersed Mice

**DOI:** 10.1007/s12035-018-0931-1

**Published:** 2018-02-10

**Authors:** Chihiro Yamada, Sachiko Mogami, Hitomi Kanno, Tomohisa Hattori

**Affiliations:** Tsumura Research Laboratories, Kampo Scientific Strategies Division, Tsumura & Co., 3586 Yoshiwara, Ami-machi, Inashiki-gun, Ibaraki, 300-1192 Japan

**Keywords:** Goal-directed behavior, Anorexia, Nest building, PYY, Dopamine

## Abstract

Apathy is observed across several neurological and psychiatric conditions; however, its pathogenesis remains unclear. We clarified the involvement of brain–gut signaling in the disruption of goal-directed behavior. Male C57BL/6J mice were exposed to water immersion (WI) stress for 3 days. Food intake and nesting behavior were measured as indexes of motivation. Repeated WI caused decrease in food intake and nesting behavior. Plasma levels of peptide YY (PYY), IL-6, and ratio of dopamine metabolites in the striatum were significantly elevated after WI. PYY and IL-6 administration significantly decreased nesting behavior. The reductions in feeding and nesting behavior were blocked by PYY receptor (Y2R) antagonist or dopamine agonist. The ameliorative effect of the Y2R antagonist was diminished by the dopamine D2 receptor (D2R) antagonist. The reduction in goal-directed behavior is associated with dysfunction of D2R signaling via increased peripheral PYY, suggesting that PYY antagonism is a novel candidate for decline of motivation in several depressive diseases.

**Electronic supplementary material:**

The online version of this article (10.1007/s12035-018-0931-1) contains supplementary material, which is available to authorized users.

## Introduction

Decreased motivation is often observed in psychoneurotic diseases with several depressive symptoms. Especially, apathy has been characterized by decreases in goal-directed behaviors and reduced motivation that frequently occurs beyond the framework of several neurological and psychiatric conditions, such as Alzheimer’s disease, major depression, and traumatic brain injury [1, 2]. In addition, eating disorders are often observed in elderly patients, particularly, among patients with these neuropsychiatric disorders. The decline in goal-directed behavior and motivation for action, which are required for life support, causes decreased physical function and increased morbidity among the elderly patients and it is an important issue in the aged society [3, 4], and efforts are under way to search for effective treatments. However, there is a lack of consensus on treatment for declined motivation, and there is no fundamental remedy for apathy [5]. Because there is no studies demonstrating initial mechanisms of apathy-like symptoms, especially with regard to decreased goal-directed behaviors.

Activation of the dopamine neurons through mesolimbic neurotransmission plays an important role in increasing motivation and reward system for critical life support behaviors. Recently, regulation of motivation for goal-directed behavior is also mediated by dopamine neurons [6]. Therefore, dopamine neurons are considered one of the candidate targets for the treatment of apathy. Accordingly, dopamine knockout (KO) mice show less basic motivation indicators such as feeding and nesting [7]. Furthermore, dopamine type 2 receptor (D2R) KO mice also exhibit hypophagia [8] and impaired goal-directed motivation [9], indicating that failure of dopamine release and/or dysfunction of the D2R play important roles in the pathogenesis of apathy-like behaviors. However, although dopamine transporter inhibitors are reportedly effective in the treatment of apathy in a small number of patients with Alzheimer’s disease [10], the effects of dopamine replacement therapy are limited and side effects pose a problem [11].

Recently, increasing evidence supports the presence of brain–gut interactions, by which peripheral hormones and the intestinal nervous system regulate central brain function. Peripheral appetite-related hormones influence feeding behavior and abnormalities of these hormone dynamics in peripheral tissues may mediate the onset of various neuropsychiatric pathologies. Peptide YY (PYY) belongs to the same family as neuropeptide Y (NPY) and pancreatic polypeptide (PP). This peptide is secreted from gastrointestinal endocrine L cells and function as neurotransmitters in central nervous system and gastrointestinal function [12]. PYY_3–36_, which is the major circulating form of PYY, predominantly activates the NPY Y2 receptor (Y2R), which is also expressed in the central nervous system and reportedly regulates neuropsychiatric mood conditions such as depression and anxiety [13–15]. Thus, it seems that activation of Y2R may be triggered decreased motivation in the neuropsychiatric disease. However, it has not been reported in relation with dopaminergic neurotransmitters and apathy symptoms.

We aimed to clarify the pathogenesis of a lack of motivation such as apathy symptoms in depressive diseases and identify targets for effective agents. In this study, we clarified whether the peripheral gastrointestinal hormone PYY is involved in the triggered induction of decreased feeding and nesting behaviors using a novel stress model induced by repeated water immersion (WI). First, we examined the influence of repeated WI on feeding and nesting behavior and determined changes in various appetite-related hormone levels in plasma and dopamine/dopamine metabolite contents in striatum. We assessed effects of the Y2R antagonist and dopamine replacement by administration the monoamine oxidase B (MAO-B) inhibitor and dopamine transporter (DAT) inhibitor. Finally, we investigated the role of D2R in this stressed model. We examined whether the decrease in goal-directed behaviors which is caused by the increase in peripheral PYY is due to the decreased function of D2R.

## Materials and Methods

### Animals

Male C57BL/6J mice of 9 weeks-of-age and 25 ± 3 g were used. They were purchased from Charles River Laboratories (Yokohama, Japan) and were housed in individual plastic cages in a room with controlled temperature and humidity under a 12-h light (23:00–11:00 h)/12-h dark cycle with free access to food and water. All experiments were performed between 08:00 and 19:00 h, and we conducted with reference to the ARRIVE guidelines. All animal care and experiments were performed in accordance with the Animal Care and Use Committee guidelines issued by the experimental animal ethics committees of Tsumura & Co. (Tokyo, Japan; permit nos. 14-065, 14-087, 15-008, 15-038, and 15-043).

### Effects of Tests Drugs on Feeding and Nesting Behaviors in WI Model or PYY Injected Mice

A mouse WI stress model was established by modifying the method which was previously described by Tanaka et al. [16]. See the Supplemental information (Table S1) for the basis of experimental conditions. Briefly, WI was applied to mice by transferring them individually to plastic cages filled with water to a depth of 5 mm (room temperature). After 14-h periods of WI, mice were returned to their original home cages. After 3 consecutive days of WI, food intake and nesting behavior were measured at beginning of the following dark phase (0 h) because nocturnal rodents are most active in the dark (experimental protocol is shown in Fig. S1).

### Evaluation of Food Intake and Nesting Behavior

Food intake was measured periodically from the onset of the dark period. Nesting behavior was evaluated every 24 h after supplying nest material (Nestlets, Animec, Tokyo, Japan) by calculating the percentages of used nest materials, and the degrees of nest completion were scored as a 5-point scale (nesting score) as reported previously [17] (Fig. S1d).

### Effects of Test Drugs on Food Intake and Nesting Behavior

Recombinant mouse IL-6 (carrier free; R&D Systems, Inc., MN, USA), human PYY_3–36_ (Bachem AG, Bubendorf, Switzerland), the Y2R antagonist BIIE0246 (Tocris Bioscience, Bristol, UK), MAO-B inhibitor pargyline hydrochloride (Sigma-Aldrich, MO, USA), DAT inhibitor methylphenidate hydrochloride (Sigma-Aldrich), and the D2R antagonist (serotonin 3 receptor antagonist) metoclopramide hydrochloride (Sigma-Aldrich) were used as test drugs. Drugs were dissolved in sterile saline and were injected in total volumes of 10 ml/kg. BIIE0246 was dissolved in 7% DMSO/saline. PYY (15–150 μg/kg) was administered via intraperitoneally (IP) injections at the onset of the dark phase (0 h) after 18 h of fasting. Subsequently, feeding and nesting behaviors were evaluated. Supplementary injections were administered at 8 h after initial injections. Pargyline (10 mg/kg) or methylphenidate (2.5 mg/kg) via IP injections were co-administered with PYY_3–36_.

We administered BIIE0246 (1.5 mg/kg), pargyline, or methylphenidate after 0, 8, and 24 h of WI and evaluated food intake and nesting behavior.

We co-administered BIIE0246 and metoclopramide (10 mg/kg) via IP injections as described.

### Measurements of Plasma Hormone Levels

Immediately after WI or 4 h after the onset of the dark period, whole blood was collected from the abdominal *vena cava* under isoflurane anesthesia. Plasma hormone concentrations were measured using the ELISA kits: AssayMax Corticosterone ELISA Kit (ASSAYPRO, MO, USA), Mouse/Rat PYY EIA Kit (Yanaihara Institute Inc., Shizuoka, Japan), Mouse IL-6 Assay Kit (IBL, Gunma, Japan), GLP-1 (Active) ELISA Kit (Shibayagi Co., Ltd., Gunma, Japan), Mouse Leptin Assay Kit (IBL, Gunma, Japan), and Mouse Metabolic Magnetic Bead Panel Kit; MMHMAG-44K (Merck Millipore, Darmstadt, Germany).

### Measurements of Hypothalamic Feeding-Related Genes

Four hours after the onset of the dark period, hypothalamus tissues were rapidly removed from mice after exsanguination under anesthesia and total RNA was extracted using RNeasy Universal Tissue kits (Qiagen, Hilden, Germany) and then reverse transcribed using TaqMan Reverse Transcription Reagents kits (Applied Biosystems, CA, USA). Quantitative PCR assays were performed using TaqMan Gene Expression Master Mix (Applied Biosystems) on a QuantStudio™ 7 Flex Real-Time PCR System (Applied Biosystems), and mRNA expression was calculated using the ΔΔCt method with oligonucleotide primers and fluorogenic probe sets for TaqMan gene-specific primer/probes (*Rps29*: Mm02342448_gH, *Npy*: Mm00445771_m1, *Agrp*: Mm00475829_g1, *Pomc*: Mm00435874_m1, *Mc4r*: Mm00457483_s1, *Crh*: Mm01293920_s1, *Il1b*: Mm01336189_m1, *Tnf*: Mm00443259_g1, *Drd2*: Mm00438545_m1, *Maob*: Mm00555412_m1, *Slc6a3*: Mm00438388_m1, *Comt*: Mm00514377_m1, Applied Biosystems).

### Measurement of Dopamine/Dopamine Metabolite Contents in Striatum Tissues

Immediately after WI, mice were exsanguinated and striatum tissues were rapidly collected and frozen. The frozen tissues were then homogenized for 30 s in perchloric acid, containing internal standard isoproterenol at 200 ng/mL, and were then incubated on ice for 30 min. The tissues were then centrifuged at 15,000 rpm for 15 min at 4 °C, and the supernatant was filtered through a 0.45-μm filter-equipped tube (UFC30HV00, Merck Millipore). The levels of dopamine and dopamine metabolites, 3,4-dihydroxyphenylacetic acid (DOPAC), and homovanillic acid (HVA), in the striatum were then determined using HPLC with electorochemical ditector (HTEC-500, Eicom Corporation, Kyoto, Japan).

### Statistical Analyses

All results are presented as means ± standard errors of the mean (SEM). Two-tailed *P* values were calculated. Pairwise differences between groups were analyzed using Student’s *t* test or Mann-Whitney *U* test. Multiple comparisons were performed using Dunnett, Steel, Tukey–Kramer, or Steel–Dwass tests. Time-dependent changes were identified using two-way repeated measure analysis of variance (ANOVA) and Bonferroni post hoc test. Differences were considered statistically significant when *P* < 0.05.

## Results

### Effects of WI on Food Intake, Body Weight, Nesting Behavior, Plasma Hormone Levels, and Hypothalamic Gene Expression

**Fig. 1:**
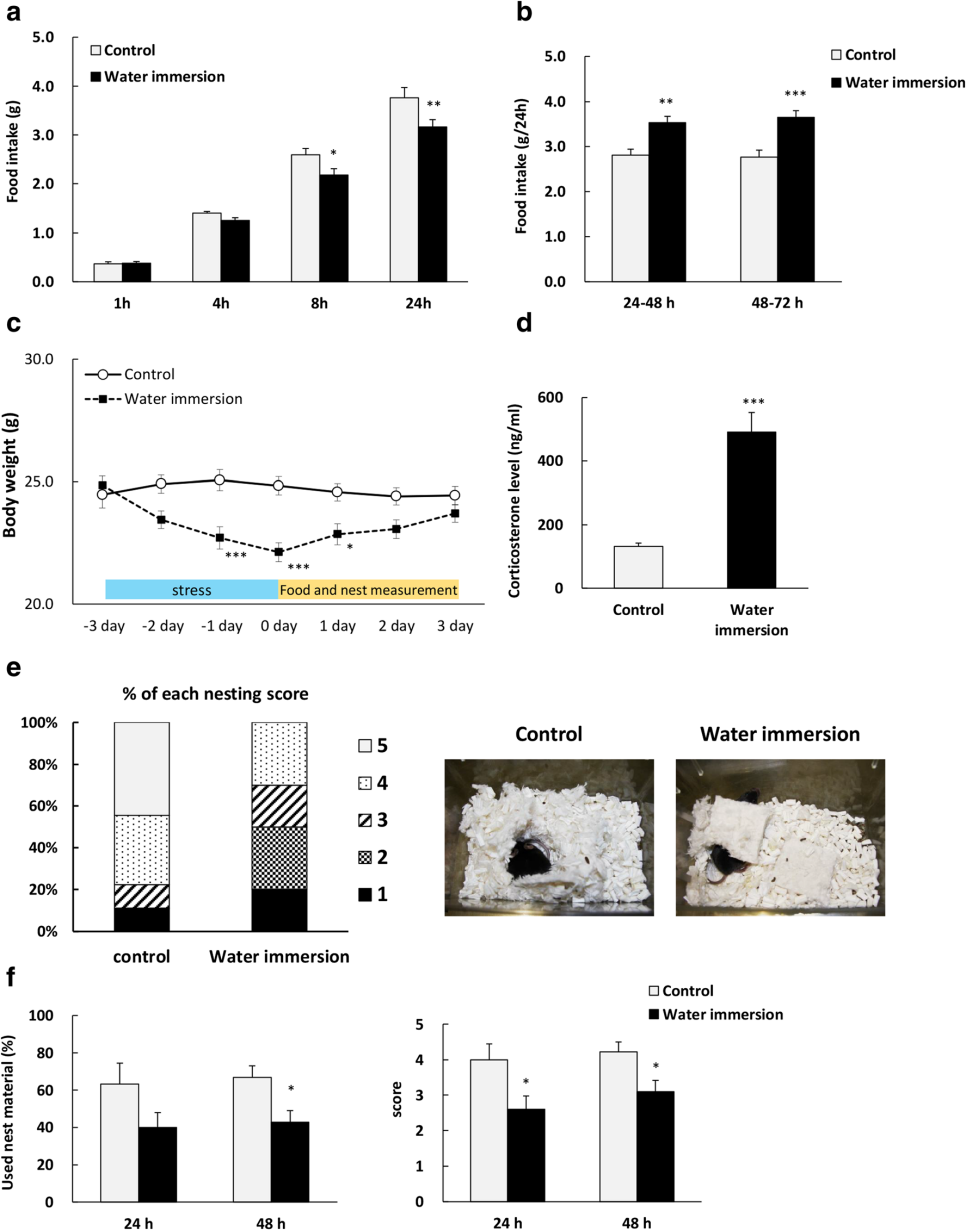
Changes in food intake, body weight, plasma corticosterone levels, and nesting behavior in water immersion mice. Effect of water immersion stress (WI, 14 h for 3 days) on **a** cumulative food intake at day 0, time × stress; *F* (3, 51) = 4.135, *P* = 0.0106, **b** 24-h cumulative food intake at days 1 and 2, **c** body weight, time × stress; *F* (6, 102) = 13.28, *P* < 0.0001, **d** plasma corticosterone levels immediately after WI, **e** the percentage of each nesting score at 1 day and a typical example of nest building at each group, and **f** nesting behavior; nesting behavior was as assessed in terms of percentages of used nesting material (%; left panel) and average of nest building score (right panel). Data represent mean ± SEM, *n* = 9–10, *, **, ***; *P* < 0.05, 0.01, 0.001 vs. control by Bonferroni, Student’s *t* test, or Mann-Whitney test

Following 3 consecutive days of 14-h WI, 24-h food intake was significantly reduced compared to the control (Fig. 1a) but was increased after 48 h (Fig. 1b). Body weight decreased significantly by WI with peak at 0 days (Fig. 1c) and plasma corticosterone levels were significantly increased (Fig. 1d) and tended to increase after 4 h (Fig. S2a). Nesting behavior was assessed by the percentages of used nest materials, and the degrees of nest completion were scored as a 5-point scale (nesting score) (Fig. S1d). Percentage of mice exhibiting high nesting score (4–5) was decreased and percentage of mice exhibiting low nesting score (1–3) was increased by WI exposure (Fig. 1e, left panel). As shown in Fig. 1e (right panel), photographs of typical nest building in control (score 5) and WI mice (score 2) were shown. The percentages of used nest materials and the average of nesting score were significantly declined at 48 h after WI (Fig. 1f).

**Fig. 2:**
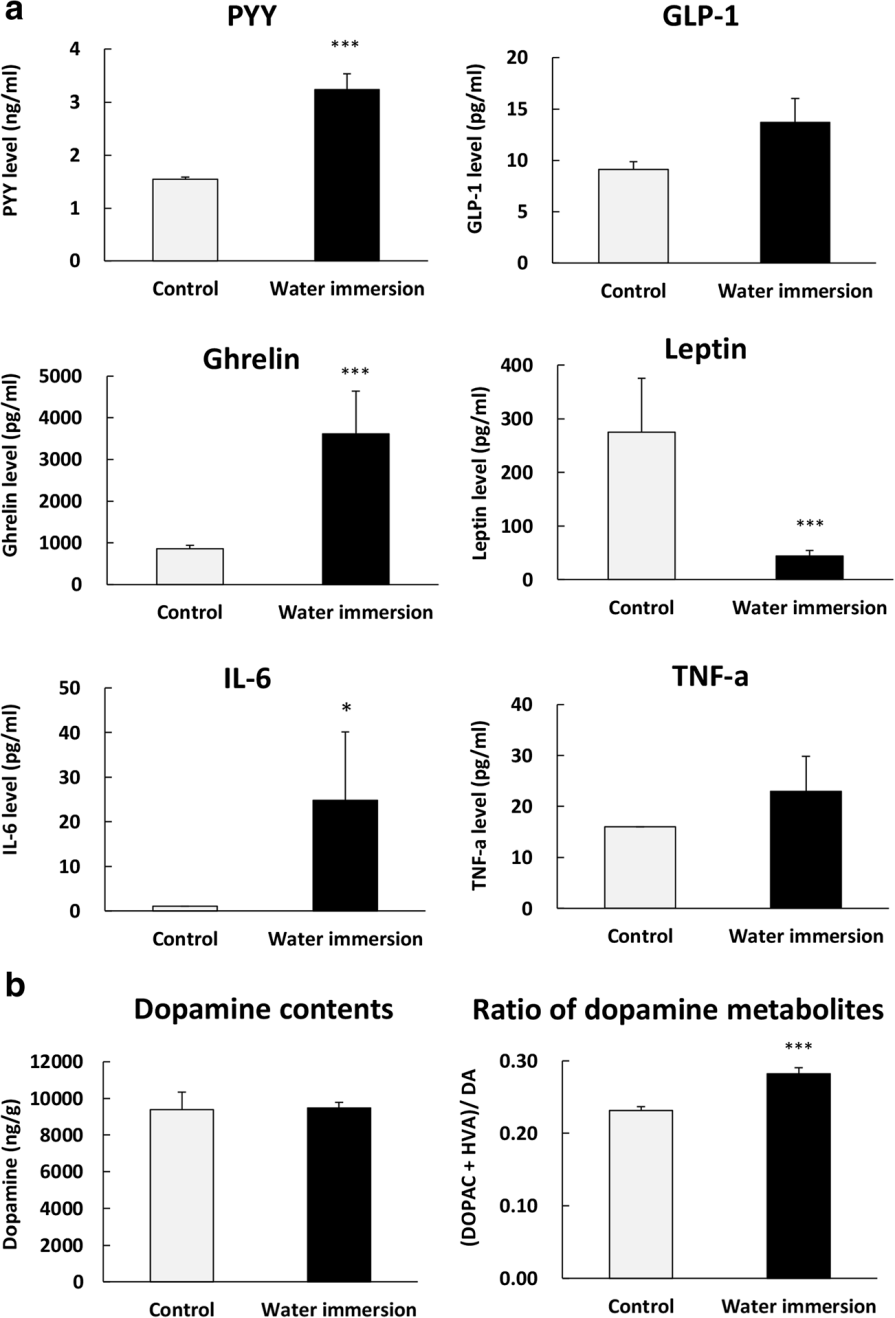
Effects of water immersion on plasma hormones and cytokines levels. **a** Plasma hormone levels at 4 h after onset of the dark period in WI mice. **b** The amount of dopamine contents and the ratio of dopamine metabolites in the striatum immediately after WI. Data represent mean ± SEM, *n* = 8–10, *, ***; *P* < 0.05, 0.001 vs. control by Student’s *t* test

To investigate the pathogenesis of anorexia in this apathy-like mouse model, we measured plasma appetite-related hormone levels and hypothalamic gene expression. Plasma levels of anorexigenic hormone, PYY, increased significantly; however, plasma levels of orexigenic ghrelin were also significantly increased by WI (Fig. 2a). In addition, plasma IL-6 levels elevated in this models. Plasma leptin levels decreased significantly and GLP-1 and TNFα levels tended to be elevated. Next, dopamine and dopamine metabolite contents in the striatum of WI-loaded mice were measured. Although dopamine contents failed to alter after WI, the ratio of DOPAC and HVA dopamine metabolites to dopamine in WI-loaded mice was significantly increased, compared with control (Fig. 2b).

*Npy* and *Agrp* mRNA expression levels in the hypothalamus were significantly increased by WI (Table 1). Moreover, expression of *Pomc* and *Mc4r* mRNA was decreased and *Crh* mRNA expression tended to increase. Expression levels of mRNAs *Il-1β* and *Tnf* were significantly increased, and that of *Slc6a3*: DAT gene tended to increase. No significant changes were observed in *Drd2*, *Maob*, and *Comt* (catechol-*O*-methyl transferase, COMT) gene mRNA expression. See Supplemental information (Table S2) for other gene expression.

**Table 1:** Gene expression in hypothalamus of water-immersed mice

Gene name	Control	Water immersion
*Npy*	1.00 ± 0.12	1.66 ± 0.18^**^
*Agrp*	1.00 ± 0.18	2.07 ± 0.30^**^
*Pomc*	1.00 ± 0.17	0.60 ± 0.07
*Mc4r*	1.00 ± 0.03	0.87 ± 0.04^*^
*Crh*	1.00 ± 0.22	1.34 ± 0.25
*Il1β*	1.00 ± 0.20	3.32 ± 0.52^**^
*Tnf*	1.00 ± 0.14	3.36 ± 0.66^**^
*Drd2*	1.00 ± 0.08	1.07 ± 0.08
*Maob*	1.00 ± 0.02	0.98 ± 0.03
*Slc6a3*	1.00 ± 0.20	1.73 ± 0.39
*Comt*	1.00 ± 0.01	1.02 ± 0.01

Gene expression in hypothalamus of WI mice was measured at 4 h after onset of the dark period; *Maob*: monoamine oxidase-B, *Slc6a3*: dopamine transporter, *Comt*: catechol-*O*-methyl transferase, *n* = 8–9; *, **; *P* < 0.05, 0.01 vs. control

In the WI mice, retroperitoneal fat mass decreased significantly (Table 2).

**Table 2:** Retroperitoneal fat weights of water-immersed mice

Control	Water immersion
0.0031 ± 0.0003 g/B.W.	0.0010 ± 0.0003 g/B.W.^***^

Retroperitoneal fat weights of WI mice were measured at 4 h after onset of the dark period. *n* = 8–9; ***; *P* < 0.001 vs. control

### Effects of Exogenous IL-6 Administration on Nesting Behavior

**Fig. 3:**
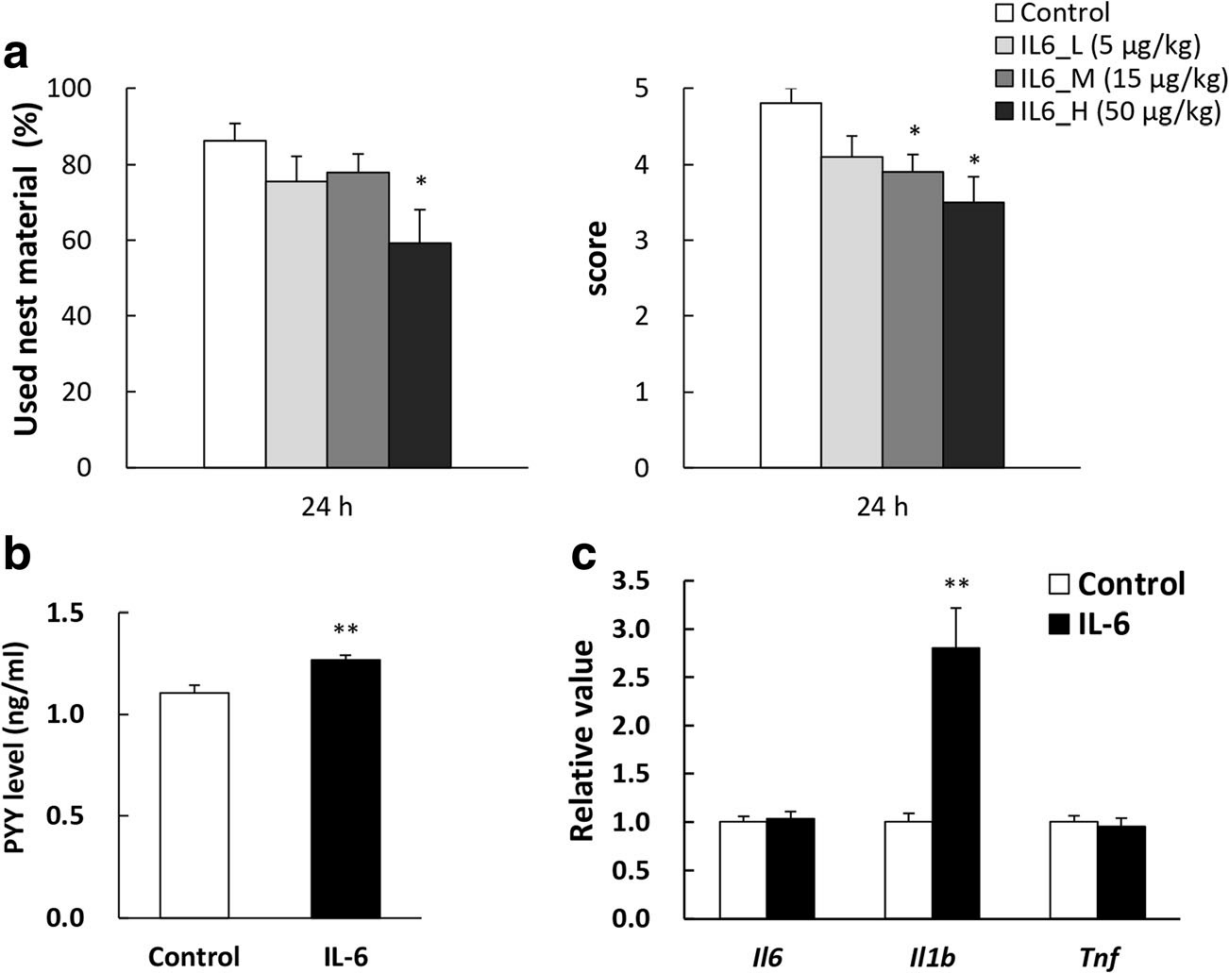
Effect of IL-6 administration in normal mice. **a** Effect of intraperitoneal (IP) injections of IL-6 (5, 15, 50 μg/kg) on nesting behavior in naive mice. **b** Effect of IL-6 (50 μg/kg, IP) on plasma PYY levels at 1 h after IP administration. **c** Effect of IL-6 (50 μg/kg, IP) on hypothalamic cytokine mRNA expression levels at 1 h after IP administration. We administered recombinant mouse IL-6 at the onset of the dark phase (0 h) after 18 h of fasting in naive mice and subsequently measured nesting behavior for 24 h, or plasma PYY levels/hypothalamic mRNA expression. Data represent mean ± SEM, *n* = 8–10, *, **; *P* < 0.05, 0.01 vs. control by Dunnett, Steel, or Student’s *t* test

IL-6 administration decreased nesting behavior and significantly increased plasma PYY levels and expression levels of mRNA *Il-1β* in the hypothalamus after 1 h at the administration (Fig. 3).

### Effects of Exogenous PYY Administration on Nesting Behavior

**Fig. 4:**
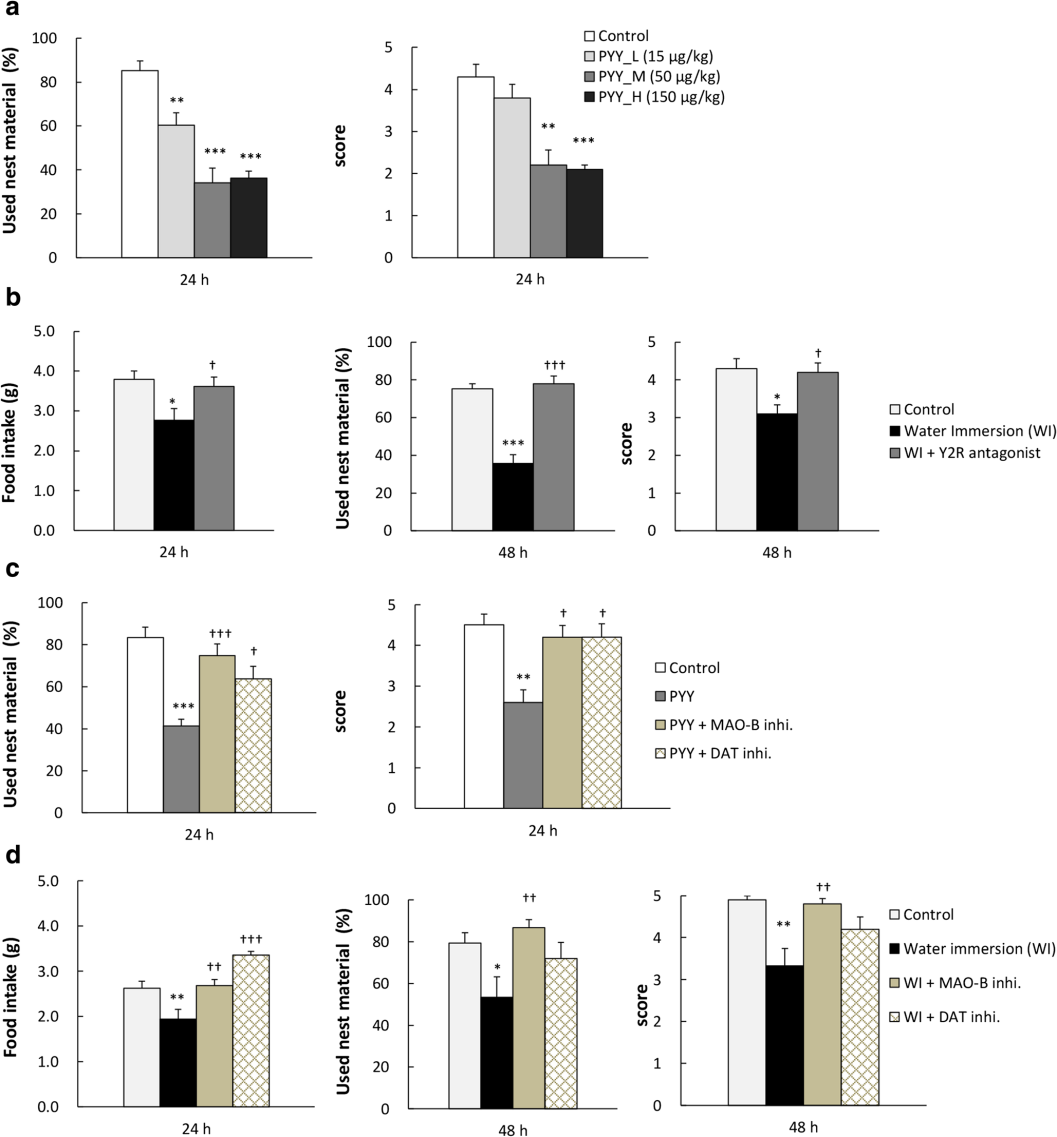
Effects of test drugs on food intake and nesting behavior in PYY injected or water immersion mice. **a** Effects of intraperitoneal (IP) injections of PYY_3–36_ (15, 50, 150 μg/kg) on nesting behavior in naive mice. **b** Effect of the Y2R antagonist BIIE0246 (1.5 mg/kg, IP) on cumulative food intake and nesting behavior in WI mice. **c** Effects of the MAO-B inhibitor pargyline (10 mg/kg, IP) or the DAT inhibitor methylphenidate (2.5 mg/kg, IP) on nesting behavior after treatment with PYY (50 μg/kg, IP). **d** Effects of the pargyline (10 mg/kg, IP) or the methylphenidate (2.5 mg/kg, IP) on cumulative food intake and nesting behavior in WI mice; Data represent mean ± SEM, *n* = 9–10, *, **, ***; *P* < 0.05, 0.01, 0.001 vs. control, †, ††, †††; *P* < 0.05, 0.01, 0.001 vs. WI or PYY by Dunnett, Steel, Tukey–Kramer, or Steel–Dwass test

PYY administration decreased nesting behavior in a dose-dependent manner (Fig. 4a) and significantly reduced food intake after 1 h at the highest concentration (Table 3). PYY administration had no effect on plasma IL-6 concentration (data not shown).

**Table 3:** One-hour cumulative food intake after PYY administration

	1-h food intake (g)
Control	0.65 ± 0.03
PYY_L (15 μg/kg)	0.58 ± 0.05
PYY_M (50 μg/kg)	0.52 ± 0.05
PYY_H (150 μg/kg)	0.45 ± 0.04^**^

Effect of intraperitoneal injections of PYY_3–36_ (15, 50, 150 μg/kg) on 1-h food intake in naive mice. *n* = 10; **; *P* < 0.01 vs. control

### Effects of Y2R Antagonist on Decreases in Food Intake and Nesting Behavior of WI Mice

To determine whether increased PYY is involved in the induction of apathy-like behaviors by WI, food intake and nesting behavior of WI mice were evaluated after IP administration of Y2R antagonist, BIIE0246 at 1.5 mg/kg (ineffective dose in naive mice, data not shown). Significant decrease in food intake after 24 h was completely blocked by the Y2R antagonist. Moreover, significantly decreased nesting behavior in WI mice was restored almost to control levels by the Y2R antagonist (Fig. 4b).

### Effects of Dopamine Supplementing Drug on Decreases in Food Intake and Nesting Behavior in PYY-Injected or WI Mice

We examined the involvement of dopaminergic neuron in the reduction of nesting behavior following PYY administration. The reduction in nesting behavior by PYY administration was significantly blocked by MAO-B or DAT inhibitors (Fig. 4c). The reduction in 2-h food intake by PYY administration was inhibited by MAO-B or DAT inhibitors (control, 0.81 ± 0.05 g; PYY, 0.52 ± 0.03 g; *P* < 0.01 vs. control; PYY + MAO-B inhibitor, 0.79 ± 0.07 g; *P* < 0.01 vs. PYY; PYY + DAT inhibitor, 0.69 ± 0.05 g; *P* = 0.09 vs. PYY). Both agents were used at their ineffective doses in naive mice (data not shown).

Both agents significantly increased the reduced food intake in WI mice (Fig. 4d). The MAO-B inhibitor significantly suppressed the reduction in nesting behavior of mice at 48 h after the final WI, whereas the DAT inhibitor had no effect (Fig. 4d). The reduction in nesting score was also significantly depressed by both agents at 24 h after the final WI (control, 4.2 ± 0.3; WI, 2.3 ± 0.4; *P* < 0.05 vs. control; WI + MAO-B inhibitor, 4.6 ± 0.3; *P* < 0.01 vs. WI, WI + DAT inhibitor, 4.6 ± 0.2; *P* < 0.01 vs. WI; as not shown in figure and tables).

### Effects of a D2R Antagonist on Restoration of Food Intake and Nesting Behavior by Y2R Antagonist Treatment in WI Mice

**Fig. 5:**
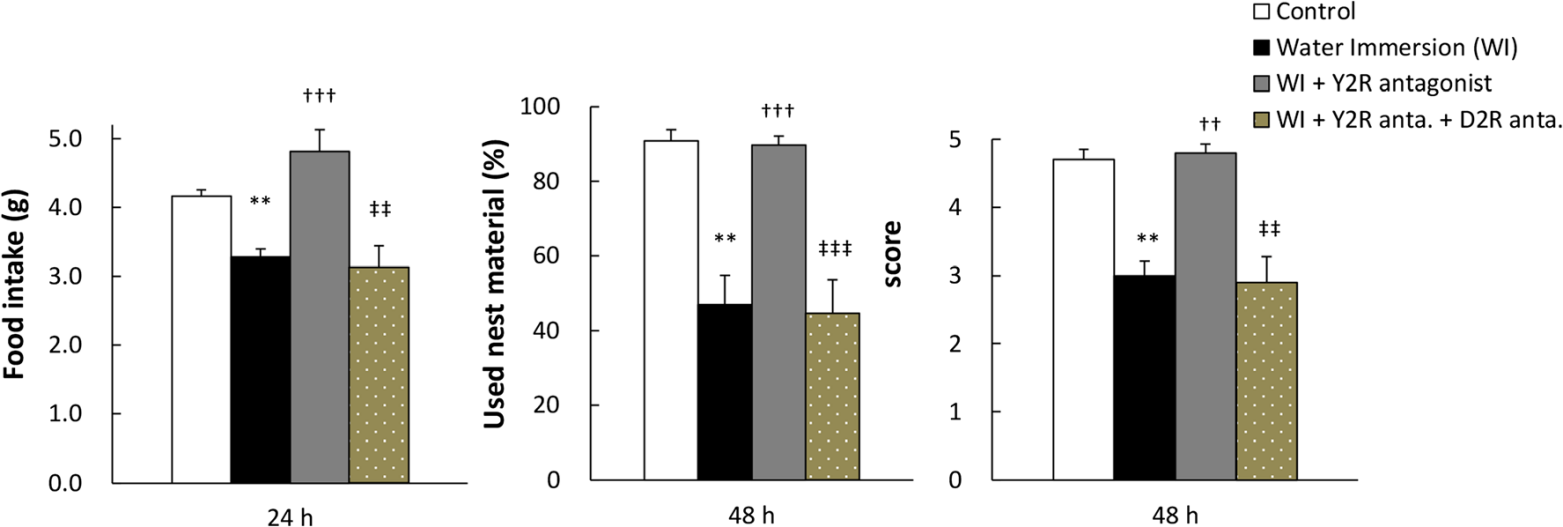
Effect of Y2R antagonist with co-administration of D2R antagonist on food intake and nesting behavior in water immersion mice. Effect of Y2R antagonist BIIE0246 (1.5 mg/kg, IP) and D2R antagonist metoclopramide (10 mg/kg, IP) on food intake and nesting behavior in WI mice; Data represent mean ± SEM, *n* = 10, **; *P* < 0.01 vs. control, ††, †††; *P* < 0.01, 0.001 vs. WI, ‡‡, ‡‡‡; *P* < 0.01, 0.001 vs. WI + Y2R antagonist by Tukey–Kramer or Steel–Dwass test

To determine whether the effects of the Y2R antagonist on food intake and nesting behavior in WI mice were mediated by D2R signaling, we performed experiments with co-administration of these agents with a D2R antagonist, metoclopramide (ineffective dose in naive mice, data not shown). We confirmed that decreased nesting behavior in WI mice was not affected by single treatment of metoclopramide (data not shown). The ameliorative effect of the Y2R antagonist in WI mice was completely blocked by the D2R antagonist (Fig. 5). These observations were also confirmed by assessment of nesting behavior.

## Discussion

In this study, repeated WI caused decreases in both feeding and nesting behaviors. Simultaneously, an increase in peripheral anorexic peptide PYY levels was observed and peripheral PYY administration induced similar phenotype as WI mice. Blockade of Y2R improved the decreased feeding and nesting behaviors. Administration of dopamine replacement drugs improved decreased feeding and nesting behaviors of PYY-injected and WI mice. Furthermore, the effect of Y2R antagonist was completely blocked by co-administration of D2R antagonist.

Evaluations of nesting behavior are one of the methods for assessing goal-directed behaviors similar to purpose accomplishment function in humans and have been used to evaluate lack of motivation in mice [17]. In previous studies, reduced nesting behavior was reported in Alzheimer’s disease and schizophrenia mice models that exhibited clear symptoms of apathy [18, 19], suggesting that nesting behavior is a useful indicator of declined motivation such as apathy symptoms. However, further studies are required to determine whether the decline in nesting behavior is characteristic of the WI mice.

We found that nesting behavior was significantly decreased after a single 14-h WI, whereas 24-h food intake was not affected (data not shown). In addition, 3 consecutive days of 6- or 10-h WI, nesting behavior was significantly reduced but food intake was not (Table S1). These results indicate that the extent of decrease in feeding behavior, which is indispensable for life support, depends on the duration of WI and the number of repetitions. Feeding behavior may be divided into behavior by homeostasis and hedonic behavior with a high preference toward foods [20]. Research on goal-directed behaviors by homeostatic feeding behavior has not been sufficiently verified. The hedonic feeding behavior clearly activates the reward system via the dopamine nervous system and enhances the motivation of goal-directed behavior [21]. Interestingly, 24–48-h food intake was increased compared to control mice, although nesting behavior was continued to diminish after 48 h. Although we found no direct evidence to show that WI load causes a hedonic feeding behavior, the activation of feeding behavior has occurred from 24 h after the end of the WI load. It is suggested that the release from WI load induces a temporary high-appetite status similar to the hedonic feeding behavior with a high motivation for feeding.

Thus, our results showed that decreased food intake occurred prior to reductions of nesting behavior. To clarify causal relationships between feeding and nesting behavior, we examined nesting behavior under restricted feeding conditions in normal mice and found that nesting behavior was inhibited depending on the extent of feeding restriction and completely reversed by re-feeding (Fig. S3). The decreased nesting behavior was associated with reduced food intake. However, nesting behavior was continued to decrease in WI mice even after the restoration of feeding behavior. These results show that lack of energy supply alone does not cause a continuous decline in nesting behavior in WI mice.

It is well known that stress causes acute stress-response and stimulates hypothalamic-pituitary-adrenal (HPA) axis. Plasma corticosterone levels, which indicate stress level in WI mice, were significantly increased immediately after final WI. However, corticosterone levels were only slightly higher than in control mice after 4 h (Fig. S2a) and were not elevated after 24 h (data not shown). This result indicates that corticosterone concentrations peak during or immediately after WI exposure. In contrast, hypophagia was sustained for 24 h, indicating that activation of HPA axis is not the dominant factor of hypophagia and decreased nesting behavior in WI mice.

Plasma ghrelin increased in WI mice compared with control mice, although leptin declined. Moreover, orexigenic gene expression of such as *Npy* and *Agrp* was significantly elevated in the hypothalamus, whereas expression levels of anorexigenic *Pomc* and *Mc4r* were diminished. The peripheral nutritional status of this model may be similar to starvation, as we also confirmed decrease in retroperitoneal fat mass. However, peripheral hunger signal is impaired by repeated WI. This condition may be similar to the ghrelin resistance as seen in cachexia [22, 23].

It was identified in this study that plasma PYY and IL-6 levels were significantly elevated in WI mice. Although IL-6 administration has no effect on food intake, IP administration of PYY_3–36_ temporally reduced food intake in agreement with the previous observations [24]. We showed for the first time that exogenous IL-6 and PYY_3–36_ administration suppressed nesting behavior in naive mice. Additionally, the treatments with exogenous administration of IL-6 significantly increased plasma PYY levels and hypothalamic *Il-1β* mRNA expression. These findings suggest that elevation of PYY or hypothalamic *Il-1β* mRNA expression in WI mice may be triggered by an increase in peripheral IL-6 production. However, the relevance of PYY and IL-6 has not been reported and further studies are required to clarify the cause of peripheral IL-6 elevation and the precise mechanism of IL-6 induced PYY in WI mice. One possibility is that bowel inflammation may be involved. Intestinal dysfunction and inflammation can be initiated by psychological stress, and the ensuing mucosal inflammation may lead to increased release of pro-inflammatory cytokines. Intestinal NPY, PYY, and PP are reported to be abnormal in patients with inflammatory bowel disease and its animal models with increased plasma IL-6 [25–27]. It is likely that interaction between these cytokines and PYY, which is derived by intestinal tissue damages, plays an important role in decreasing feeding and nesting behaviors. The promoting effect of exogenous IL-6 administration on PYY release was obviously significant but minimal, which may be because a single administration of IL-6 will only cause transit IL-6 levels in the blood but cannot maintain high concentration as seen in WI mice.

We showed that peripheral administration of Y2R antagonist significantly restored the decreased food intake and nesting behavior in WI mice to the control levels. These results suggest that the activated Y2R is a key mediator for lack of motivation such as apathy-like symptoms. Previous studies have confirmed that activation of the Y2R is related to social interaction disorders [28] and the onset of depression-like symptoms [29]. In addition, Y2R KO mice exhibited anti-anxiety and anti-depressed phenotype in elevated plus-maze, open field, and forced swimming tests [30], and administration of Y2R antagonist led to anti-depressant effects [31]. These findings partially support a hypothesis that elevated peripheral PYY may lead to decrease in motivation in WI mice. However, the precise mechanism for the effect of peripheral PYY is still not completely understood. A direct action of circulating PYY through Y2R located in specific brain regions, including the arcuate nucleus, has been proposed to be involved in the regulation of food intake. On the other hand, since the anorexic effect due to PYY administration is blocked by vagotomy [32], it may be transmitted by the vagal afferents emerging from Y2R located in the gut. The effect of PYY in WI mice was thought to be either direct action on the brain or via the vagus nerve. It is well known that Y2R exists on the NPY presynaptic neurons and plays a role as an autoreceptor for NPY synthesis. Therefore, it is possible that NPY also acts on Y2R autoreceptors. However, in this study, because the NPY mRNA expression in the hypothalamus of the WI mice was significantly increased, the possibility that PYY and NPY acted on Y2R on the NPY neurons is small. Immunohistochemical studies have revealed that Y2R is widely localized in the brain, such as in the brain stem area, nucleus accumbens, amygdala, hippocampus, hypothalamus, substantia nigra compacta, and nucleus tractus solitarius [33]. In this study, we could not completely eliminate the relationship between increased NPY due to WI load and Y2R activation. Meanwhile, peripheral PYY injection acts on the arcuate nucleus as well as the nucleus tractus solitarius and area postrema [34]. Effects of the systemically administered Y2R agonist may also be directly exerted at the dorsal vagal complex in the medulla [35]. Therefore, it seems likely that an increase in peripheral PYY may be involved in the activation of Y2R in these brain areas.

PYY_3–36_ has been reported to modulate dopamine release. Stadlbauer and Batterham et al. demonstrated that PYY enhances the action of dopamine under novel object exploration and acute dopaminergic drug challenge conditions [28, 36, 37]. Brunetti et al. have demonstrated that PYY inhibits dopamine release in the hypothalamus [38], and Ault et al. have concluded using rat nucleus accumbens that PYY does not affect dopamine release [39]. These inconsistent results imply that the influence of PYY on dopamine release greatly varies depending on the experimental condition, and the discussion remains controversial.

**Fig. 6:**
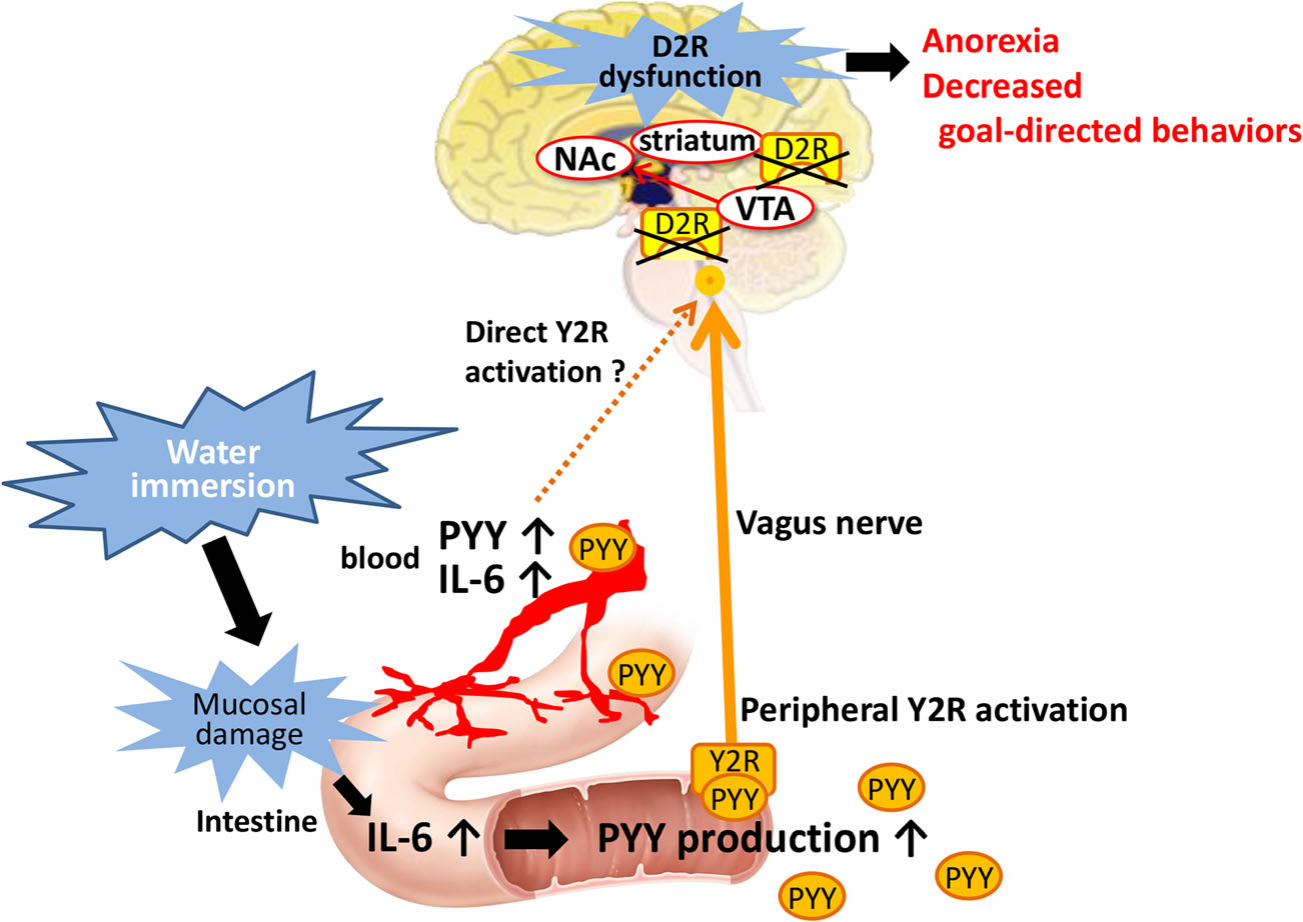
Hypothetical schema of onset mechanisms of apathy-like phenotype such as anorexia and decreased goal-directed behavior in water-immersed mice. Water immersion induces peripheral IL-6 and PYY elevation which may lead to D2R dysfunction. D2R dysfunction in the reward system is considered to be involved in the induction of anorexia and decreased goal-directed behavior

In this study, administration of MAO-B or DAT inhibitors to WI mice or PYY injection mice significantly ameliorated feeding and nesting behaviors. We hypothesized that increased peripheral PYY activated the Y2R which causes decreased dopamine release and/or dysfunction of dopamine receptor in the brain, leading to the decrease in motivation (Fig. 6). In partial agreement with our hypothesis, regulation of motivation for goal-directed behavior is mediated by dopamine neurons projecting from the substantia nigra to the striatum [6]. Nesting behavior of dopamine deficient mice was significantly decreased and reversed by L-DOPA treatment [7]. Additionally, previous study showed that PYY inhibits dopamine release from rat hypothalamic tissues [38]. However, because Y2R stimulation reportedly promoted synthesis of dopamine precursors in striatal tissues from rats [40], the precise mechanisms remain controversial. It is reportedly suggested that regulated release of dopamine in the dorsal striatum is essential for normal feeding and, probably, many other goal-directed behaviors [6, 41]. The ratios of dopamine metabolites to dopamine were significantly elevated in WI mice. Abnormalities in dopamine metabolism and suppression of dopamine production were previously observed in a chronic WI rat model [16, 42]. Hence, abnormalities in dopamine metabolism may also have occurred in WI mice. The parkinsonian striatum tissues showed an increased dopamine turnover relative to the control striatum tissues [43], and both DOPAC and HVA cerebrospinal fluid levels were increased with motor impairments in patients with Parkinson’s disease [44]. Therefore, an elevated dopamine turnover may influence feeding and nesting behaviors of WI mice. However, because we did not determine the amount of dopamine release in this study, further perfusion and functional studies on D2R expression and dopamine release are required.

Meanwhile, ghrelin transmits the signal to brain via the vagus nerve and enhances the dopamine signal from the ventral tegmental area (VTA) to the nucleus accumbens (NAc) [45]. In WI mice, plasma ghrelin was increased, and the expression of *Npy*/*Agrp* gene in the hypothalamus was also significantly increased compared to the control group. Nevertheless, feeding and nesting behaviors remained decreased suggesting one possibility that PYY might be counteracting the effect of ghrelin related to dopamine signal activation.

Central dopamine D1 and D2 receptors are critical mediators of reward signaling. The food reward circuit is reported to be associated with D2R expression and function in the striatum and NAc [20]. Moreover, other studies show that D2R KO mice exhibit anorexia status [8], disorder of goal-directed motivation [9], and blockade of D2R reduces feeding [46] and nesting behavior [47], suggesting that D2R activation significantly affects particularly in the motivation for feeding and nesting behaviors. Herein, we demonstrated that the ameliorative effects of the Y2R antagonist on feeding and nesting behaviors in WI mice were almost completely blocked by the D2R antagonist. This result also supports our hypothesis. Further investigations are needed to clarify the mechanisms of dopamine signal dysfunction via D2R by peripheral PYY.

Although it is the basic research, we could clarify the development of a rodent model with symptoms similar to apathy and identified the mechanism candidates useful for its treatment. Therefore, it is desirable to conduct well-controlled clinical trials of Y2 receptor antagonist on apathy symptoms in dementia or Parkinson’s disease.

In conclusion, repeated WI reduced feeding and nesting behaviors in mice and led to a lack of motivation, such as apathy-like symptoms. This phenotype was found to be caused by impaired dopamine signaling through D2R induced by peripheral PYY elevation in this model. The present study indicates the potential of Y2R antagonist to be new effective treatment for anorexia and loss of motivation.

## Electronic Supplementary Material

ESM 1 (PDF 287 kb)

## References

[1] Bonnelle V, Veromann KR, Burnett Heyes S, Lo Sterzo E, Manohar S, Husain M (2015). Characterization of reward and effort mechanisms in apathy. J Physiol Paris.

[2] Robert PH, Mulin E, Mallea P, David R (2010). REVIEW: apathy diagnosis, assessment, and treatment in Alzheimer’s disease. CNS Neurosci Ther.

[3] Rowe JW, Kahn RL (1987). Human aging: usual and successful. Science.

[4] Seeman TE, Robbins RJ (1994). Aging and hypothalamic-pituitary-adrenal response to challenge in humans. Endocr Rev.

[5] Ishizaki J, Mimura M (2011). Dysthymia and apathy: diagnosis and treatment. Depression Res Treat.

[6] Baik JH (2013). Dopamine signaling in food addiction: role of dopamine D2 receptors. BMB Rep.

[7] Szczypka MS, Kwok K, Brot MD, Marck BT, Matsumoto AM, Donahue BA, Palmiter RD (2001). Dopamine production in the caudate putamen restores feeding in dopamine-deficient mice. Neuron.

[8] Kim KS, Yoon YR, Lee HJ, Yoon S, Kim SY, Shin SW, An JJ, Kim MS, Choi SY, Sun W, Baik JH (2010). Enhanced hypothalamic leptin signaling in mice lacking dopamine D2 receptors. J Biol Chem.

[9] Tsutsui-Kimura I, Takiue H, Yoshida K, Xu M, Yano R, Ohta H, Nishida H, Bouchekioua Y, Okano H, Uchigashima M, Watanabe M, Takata N, Drew MR, Sano H, Mimura M, Tanaka KF (2017). Dysfunction of ventrolateral striatal dopamine receptor type 2-expressing medium spiny neurons impairs instrumental motivation. Nat Commun.

[10] Rosenberg PB, Lanctot KL, Drye LT, Herrmann N, Scherer RW, Bachman DL, Mintzer JE, Investigators A (2013). Safety and efficacy of methylphenidate for apathy in Alzheimer’s disease: a randomized, placebo-controlled trial. J Clin Psychiatry.

[11] Rea R, Carotenuto A, Fasanaro AM, Traini E, Amenta F (2014). Apathy in Alzheimer’s disease: any effective treatment?. TheScientificWorldJOURNAL.

[12] Holzer P, Reichmann F, Farzi A (2012). Neuropeptide Y, peptide YY and pancreatic polypeptide in the gut-brain axis. Neuropeptides.

[13] Redrobe JP, Dumont Y, Herzog H, Quirion R (2003). Neuropeptide Y (NPY) Y2 receptors mediate behaviour in two animal models of anxiety: evidence from Y2 receptor knockout mice. Behav Brain Res.

[14] Tschenett A, Singewald N, Carli M, Balducci C, Salchner P, Vezzani A, Herzog H, Sperk G (2003). Reduced anxiety and improved stress coping ability in mice lacking NPY-Y2 receptors. Eur J Neurosci.

[15] Heilig M (2004). The NPY system in stress, anxiety and depression. Neuropeptides.

[16] Tanaka M, Nakamura F, Mizokawa S, Matsumura A, Nozaki S, Watanabe Y (2003). Establishment and assessment of a rat model of fatigue. Neurosci Lett.

[17] Yan L, Li L, Han W, Pan B, Xue X, Mei B (2013). Age-related neuropsychiatric symptoms in presenilins conditional double knockout mice. Brain Res Bull.

[18] Filali M, Lalonde R, Rivest S (2009). Cognitive and non-cognitive behaviors in an APPswe/PS1 bigenic model of Alzheimer’s disease. Genes Brain Behav.

[19] Pedersen CS, Sorensen DB, Parachikova AI, Plath N (2014). PCP-induced deficits in murine nest building activity: employment of an ethological rodent behavior to mimic negative-like symptoms of schizophrenia. Behav Brain Res.

[20] Baik JH (2013). Dopamine signaling in reward-related behaviors. Front Neural Circ.

[21] Meye FJ, Adan RA (2014). Feelings about food: the ventral tegmental area in food reward and emotional eating. Trends Pharmacol Sci.

[22] Fujitsuka N, Asakawa A, Uezono Y, Minami K, Yamaguchi T, Niijima A, Yada T, Maejima Y, Sedbazar U, Sakai T, Hattori T, Kase Y, Inui A (2011). Potentiation of ghrelin signaling attenuates cancer anorexia-cachexia and prolongs survival. Transl Psychiatry.

[23] Terawaki K, Sawada Y, Kashiwase Y, Hashimoto H, Yoshimura M, Suzuki M, Miyano K, Sudo Y, Shiraishi S, Higami Y, Yanagihara K, Kase Y, Ueta Y, Uezono Y (2014). New cancer cachexia rat model generated by implantation of a peritoneal dissemination-derived human stomach cancer cell line. Am J Physiol Endocrinol Metab.

[24] Stadlbauer U, Woods SC, Langhans W, Meyer U (2015). PYY3-36: beyond food intake. Front Neuroendocrinol.

[25] El-Salhy M, Hausken T (2016). The role of the neuropeptide Y (NPY) family in the pathophysiology of inflammatory bowel disease (IBD). Neuropeptides.

[26] Hassan AM, Jain P, Reichmann F, Mayerhofer R, Farzi A, Schuligoi R, Holzer P (2014). Repeated predictable stress causes resilience against colitis-induced behavioral changes in mice. Front Behav Neurosci.

[27] Funderburg NT, Stubblefield Park SR, Sung HC, Hardy G, Clagett B, Ignatz-Hoover J, Harding CV, Fu P, Katz JA, Lederman MM, Levine AD (2013). Circulating CD4(+) and CD8(+) T cells are activated in inflammatory bowel disease and are associated with plasma markers of inflammation. Immunology.

[28] Stadlbauer U, Langhans W, Meyer U (2013). Administration of the Y2 receptor agonist PYY3-36 in mice induces multiple behavioral changes relevant to schizophrenia. Neuropsychopharmacology.

[29] Morales-Medina JC, Dumont Y, Quirion R (2010). A possible role of neuropeptide Y in depression and stress. Brain Res.

[30] Carvajal C, Dumont Y, Herzog H, Quirion R (2006). Emotional behavior in aged neuropeptide Y (NPY) Y2 knockout mice. J Mol Neurosci: MN.

[31] Redrobe JP, Dumont Y, Fournier A, Quirion R (2002). The neuropeptide Y (NPY) Y1 receptor subtype mediates NPY-induced antidepressant-like activity in the mouse forced swimming test. Neuropsychopharmacology.

[32] Abbott CR, Monteiro M, Small CJ, Sajedi A, Smith KL, Parkinson JR, Ghatei MA, Bloom SR (2005). The inhibitory effects of peripheral administration of peptide YY(3-36) and glucagon-like peptide-1 on food intake are attenuated by ablation of the vagal-brainstem-hypothalamic pathway. Brain Res.

[33] Stanic D, Brumovsky P, Fetissov S, Shuster S, Herzog H, Hokfelt T (2006). Characterization of neuropeptide Y2 receptor protein expression in the mouse brain. I. Distribution in cell bodies and nerve terminals. J Comp Neurol.

[34] Blevins JE, Chelikani PK, Haver AC, Reidelberger RD (2008). PYY(3-36) induces Fos in the arcuate nucleus and in both catecholaminergic and non-catecholaminergic neurons in the nucleus tractus solitarius of rats. Peptides.

[35] Parker SL, Balasubramaniam A (2008). Neuropeptide Y Y2 receptor in health and disease. Br J Pharmacol.

[36] Batterham RL, ffytche DH, Rosenthal JM, Zelaya FO, Barker GJ, Withers DJ, Williams SC (2007). PYY modulation of cortical and hypothalamic brain areas predicts feeding behaviour in humans. Nature.

[37] Stadlbauer U, Weber E, Langhans W, Meyer U (2014). The Y2 receptor agonist PYY(3-36) increases the behavioural response to novelty and acute dopaminergic drug challenge in mice. Int J Neuropsychopharmacol.

[38] Brunetti L, Orlando G, Ferrante C, Chiavaroli A, Vacca M (2005). Peptide YY (3 -36) inhibits dopamine and norepinephrine release in the hypothalamus. Eur J Pharmacol.

[39] Ault DT, Werling LL (1998). Neuropeptide Y-mediated enhancement of NMDA-stimulated [3H]dopamine release from rat prefrontal cortex is reversed by sigma1 receptor antagonists. Schizophr Res.

[40] Adewale AS, Macarthur H, Westfall TC (2005). Neuropeptide Y induced modulation of dopamine synthesis in the striatum. Regul Pept.

[41] Sotak BN, Hnasko TS, Robinson S, Kremer EJ, Palmiter RD (2005). Dysregulation of dopamine signaling in the dorsal striatum inhibits feeding. Brain Res.

[42] Ogawa T, Shishioh-Ikejima N, Konishi H, Makino T, Sei H, Kiryu-Seo S, Tanaka M, Watanabe Y, Kiyama H (2009). Chronic stress elicits prolonged activation of alpha-MSH secretion and subsequent degeneration of melanotroph. J Neurochem.

[43] Ogawa N, Tanaka K, Asanuma M (2000). Bromocriptine markedly suppresses levodopa-induced abnormal increase of dopamine turnover in the parkinsonian striatum. Neurochem Res.

[44] Stefani A, Pierantozzi M, Olivola E, Galati S, Cerroni R, D'Angelo V, Hainsworth AH, Saviozzi V, Fedele E, Liguori C (2017). Homovanillic acid in CSF of mild stage Parkinson’s disease patients correlates with motor impairment. Neurochem Int.

[45] Perello M, Dickson SL (2015). Ghrelin signalling on food reward: a salient link between the gut and the mesolimbic system. J Neuroendocrinol.

[46] Baldo BA, Sadeghian K, Basso AM, Kelley AE (2002). Effects of selective dopamine D1 or D2 receptor blockade within nucleus accumbens subregions on ingestive behavior and associated motor activity. Behav Brain Res.

[47] Silva MR, Bernardi MM, Felicio LF (2001). Effects of dopamine receptor antagonists on ongoing maternal behavior in rats. Pharmacol Biochem Behav.

