# Supplementary material for: Peptide YY Causes Apathy-Like Behavior via the Dopamine D2 Receptor in Repeated Water-Immersed Mice
Source: Mol Neurobiol. 2018 Feb 10;55(9):7555–66. doi: 10.1007/s12035-018-0931-1 (PMC6096978; doi:10.1007/s12035-018-0931-1)
Supplement: Supplementary file 1 — (PDF 287 kb) [file 12035_2018_931_MOESM1_ESM.pdf]

Peptide YY causes apathy-like behavior via the dopamine D2 receptor in repeated water immersed mice, Mol Neurobiol, Yamada C.; Tsumura & Co., hattori_tomohisa@mail.tsumura.co.jp

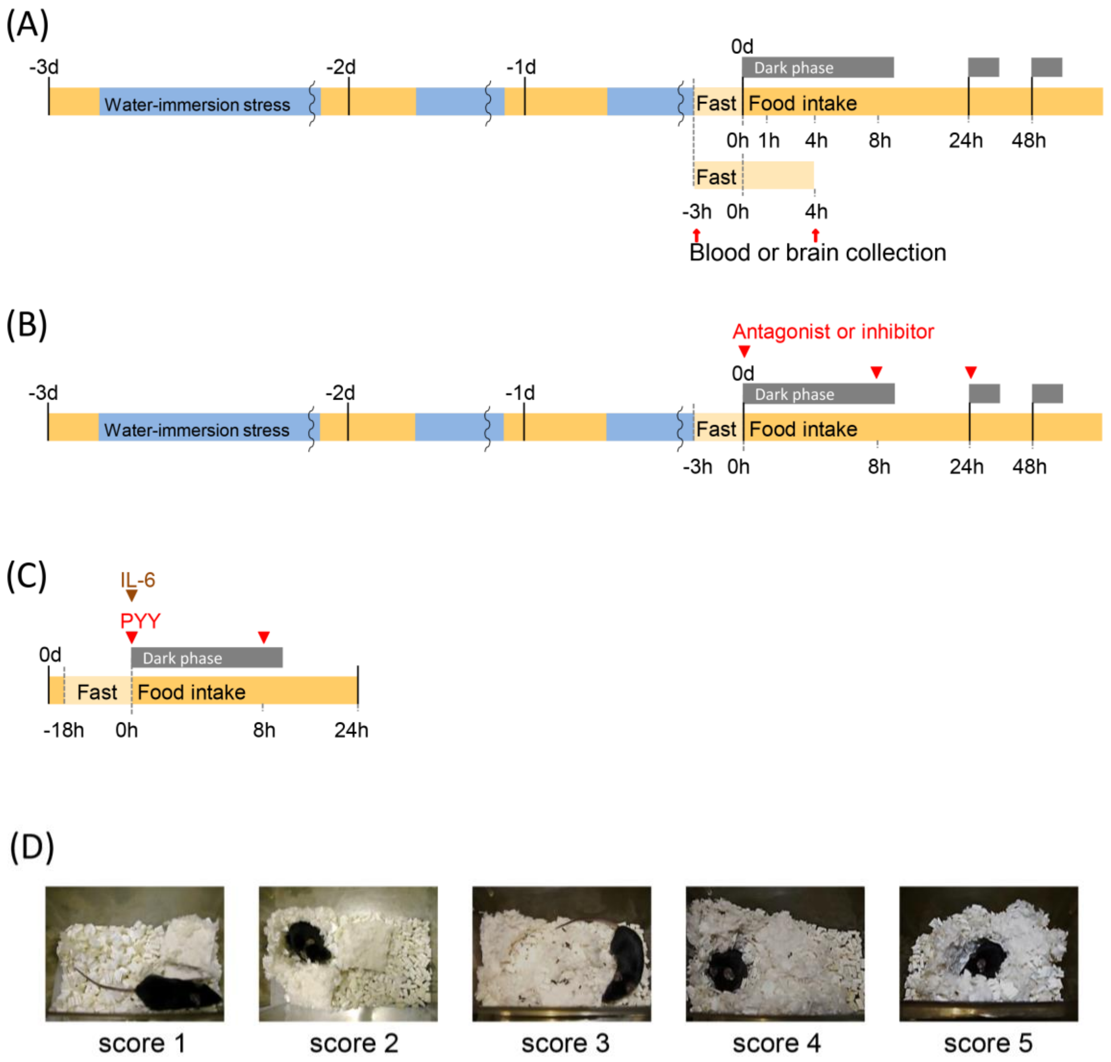

Supplementary figure S1 Experimental protocol

(A) Experimental design of water immersion mice. Water immersion stress was applied to the mice for 14 h and 3 consecutive days. We started to measure food intake and nesting behavior at the beginning of the following dark phase after the final immersion. (B) Experimental design for investigating the effects of Y2 receptor antagonist (BIIE0246, 1.5 mg/kg, IP), MAO-B inhibitor (Pargyline, 10 mg/kg, IP) or DAT inhibitor (Methylphenidate, 2.5 mg/kg, IP) in water immersion mice. (C) Administration of PYY_{3-36} (15, 50, 150 μg/kg, IP) or IL-6 (5, 15, 50 μg/kg, IP) to normal mice. (D) A typical example of nest building at each nesting score.

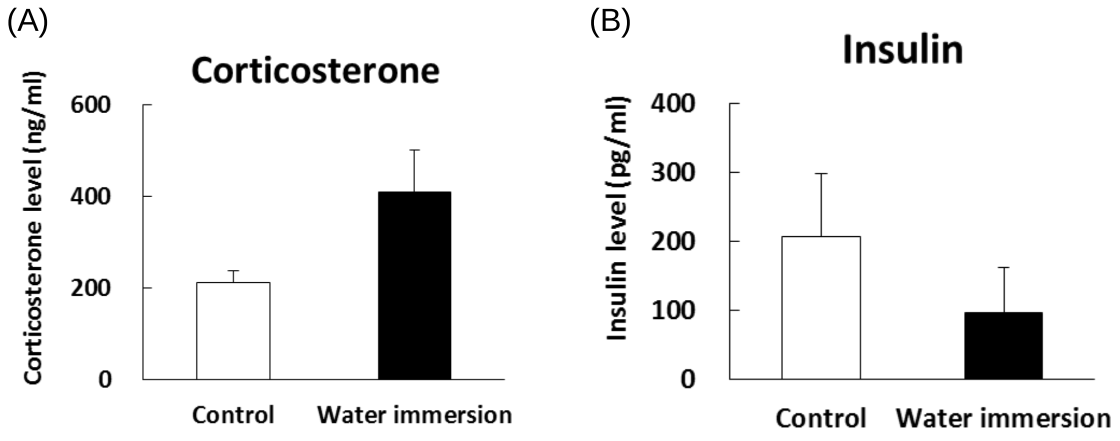

Supplementary figure S2 Plasma hormone levels in water immersion mice

Plasma corticosterone (A) and insulin (B) levels at 4 h after the onset of the dark phase in water immersion mice.

Plasma hormone concentrations were measured by using the methods in Figure 2 information.

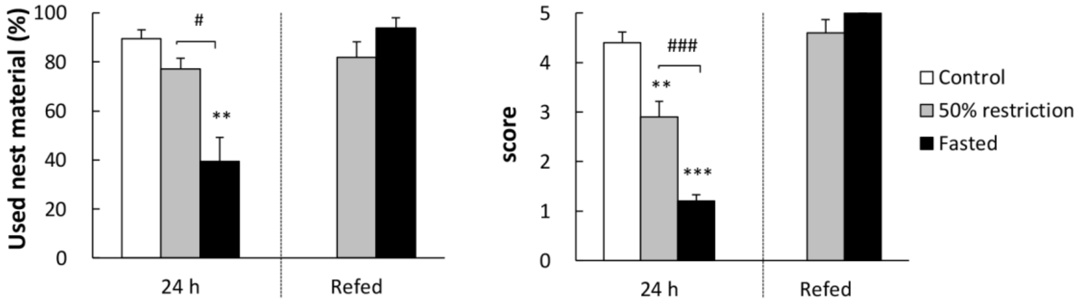

Supplementary figure S3 Effect of food consumption on nesting behavior in normal mice

Nesting behavior was evaluated for 24 h after supplying nesting materials to investigate the relations with feeding behaviors in normal mice. Fifty percent restriction group mice were fed with 50% amount of food compared to the control mice from the previous day. Fasted mice were supplied with nesting materials after 16 h food deprivation. After the 24 h evaluation, 50% restriction group and fasted group were fed *ad libitum* (refed) and nesting behavior was evaluated for 24 h. **, ***; $P < 0.01, 0.001$ vs. control, #, ###; $P < 0.05, 0.001$.

Supplementary table S1 Influences of water immersion time on feeding and nesting behaviors in mice

	Food intake (g)	Used nest material (%)	
	24 h	24 h	48 h
Control	2.97 ± 0.12	88.4 ± 4.8	94.4 ± 2.5
Water immersion_ 6h×3days	2.94 ± 0.10	77.6 ± 4.7	96.8 ± 1.3
Water immersion_10h×3days	2.87 ± 0.10	46.2 ± 6.3***	67.6 ± 6.0**

, *; $P < 0.01, 0.001$ vs. control.

We did not observe any significant changes in 24-h food intake and nesting behavior by 3 consecutive days of 6-h water immersion. By 3 consecutive days of 10-h water immersion, 24-h food intake tended to decline and nesting behavior was significantly reduced.

Supplementary table S2 Gene expression in hypothalamus of water immersed mice

Gene name	Control	Water immersion	*P* values
Ghrl	1.00 ± 0.06	0.83 ± 0.07	n.s.
Ghsr	1.00 ± 0.06	1.01 ± 0.08	n.s.
Lepr	1.00 ± 0.12	1.23 ± 0.05	n.s.
Hcrt	1.00 ± 0.07	1.06 ± 0.09	n.s.
Gcg	1.00 ± 0.33	0.90 ± 0.12	n.s.
Glp1r	1.00 ± 0.02	1.07 ± 0.06	n.s.
Il6	1.00 ± 0.10	0.73 ± 0.10	n.s.
Tgfb1	1.00 ± 0.03	1.11 ± 0.04	n.s.

Gene expression in hypothalamus of water immersed mice was measured at 4 h after the onset of the dark phase.
